# The Cyclopedia of Practical Medicine

**Published:** 1844-12

**Authors:** 


*The Cyclopædia of Practical Medicine.*—The first number of this work is in our possession, and fully sustains its character and title of being a practical work.

Its subject matter is arranged alphabetically, and is treated on by men of the highest talent, standing, and practical experience in the medical profession.

It is edited by Drs. Forbes, Twedie, and Conolly. The American edition is published by Lea and Blanchard. Revised and with additions by Professor Dungleson. From such men and the extent of the work, embracing, as it does every thing valuable, in the whole circle of practical medicine. The medical community will thus have an opportunity of furnishing themselves with one of the most comprehensive publications known to the profession.

It will be completed in twenty-four parts, at 50 cents a part.

This work, however, is not only necessary and useful to the physician, but it also contains information that should be possessed by every dentist, and it is for the benefit of this last class of practitioners that this notice is more especially intended, and we most earnestly invite the attention of our professional brethren to it.

The science of Dentistry is now acknowledged to be a necessary and essential part of the science of medicine. That it is the link—and an essential link in the great chain of medicine for preserving the health and relieving the sufferings of our afflicted fellow beings. And that it cannot hence be studied by and of itself, apart and independent of the other branches of medicine.

But further, the science of Dental Surgery is now zealously and industriously striving for an elevated stand; yes, for an equal stand, which is no more than her right, among the sister sciences. She is making noble efforts in every part of our country for the elevation of the profession—for raising it from the degraded condition which it has too long occupied, and placing its members, by the light of scientific medical knowledge, on an equality with the other learned professions.

To this most desirable end, it gives us pleasure to report the fact, several times announced before in our Journal, and which, we humbly think, cannot too frequently be brought to the public eye, as well as before the profession itself, which is, the establishment of a college for the express purpose of educating and practically teaching young men, who shall thus go out suitably qualified to credit to it and themselves, honor to their profession and lasting benefit to the community.

